# Multimaterial 3D laser microprinting using an integrated microfluidic system

**DOI:** 10.1126/sciadv.aau9160

**Published:** 2019-02-08

**Authors:** Frederik Mayer, Stefan Richter, Johann Westhauser, Eva Blasco, Christopher Barner-Kowollik, Martin Wegener

**Affiliations:** 1 Institute of Nanotechnology (INT), Karlsruhe Institute of Technology (KIT), 76128 Karlsruhe, Germany.; 2 Institute of Applied Physics (APH), Karlsruhe Institute of Technology (KIT), 76128 Karlsruhe, Germany.; 3 Carl Zeiss AG, Carl Zeiss Promenade 10, 07745 Jena, Germany.; 4 Macromolecular Architectures, Institute of Technical Chemistry and Polymer Chemistry (ITCP), Karlsruhe Institute of Technology (KIT), 76128 Karlsruhe, Germany.; 5 School of Chemistry, Physics and Mechanical Engineering, Queensland University of Technology (QUT), 2 George Street, Brisbane, QLD 4000, Australia.

## Abstract

Three-dimensional (3D) laser micro- and nanoprinting has become a versatile, reliable, and commercially available technology for the preparation of complex 3D architectures for diverse applications. However, the vast majority of structures published so far have been composed of only a single constituent material. Here, we present a system based on a microfluidic chamber integrated into a state-of-the-art laser lithography apparatus. This system is scalable in terms of the number of materials and eliminates the need to go back and forth between the lithography instrument and the chemistry room numerous times, with tedious realignment steps in between. As an application, we present 3D deterministic microstructured security features requiring seven different liquids: a nonfluorescent photoresist as backbone, two photoresists containing different fluorescent quantum dots, two photoresists with different fluorescent dyes, and two developers. Our integrated microfluidic 3D printing system opens the door to truly multimaterial 3D additive manufacturing on the micro- and nanoscale.

## Teaser

An instrument brings 3D laser printing to a new level, exemplified by 3D fluorescent microstructures composed of five materials.

## INTRODUCTION

Three-dimensional (3D) laser micro- and nanoprinting, previously often referred to as 3D direct laser writing, has turned from an interesting scientific curiosity that emerged around 20 years ago (*1*, *2*) into a reliable, versatile, commercially available, and widespread technology. Applications range from 3D photonic crystals (*3*, *4*), photonic wire bonds (*5*), 3D-printed free-form surfaces (*6*, *7*), microoptics for 3D optical circuitry (*8*), micromirrors (*9*), and optical microlens systems (*10*, *11*) via 3D mechanical metamaterials (*12*–*15*) and 3D security features (*16*) to 3D microscaffolds for cell culture (*17*–*19*) and 3D-printed micromachines (*20*–*24*). However, in the vast majority of published microstructures, only a single material has been 3D-printed, with several recent notable exceptions (*7*, *16*, *25*, *26*). The state of the art of this field has been described in various recent reviews (*27*–*29*), where further references can be found.

In close analogy to well-established multistep planar optical lithography, 3D structures composed of multiple materials are straightforward to make by the application of the first resist (e.g., by drop casting or spin coating), exposing the first resist system by two-photon absorption, development and rinsing in the chemistry room, application of the next resist, realignment in three dimensions within the 3D laser printer, exposing the second resist system, etc. However, as resist systems and cycles increase, such a process performed by humans rapidly becomes not only very tedious and time consuming but also quite unreliable. Therefore, it is highly desirable to avoid having to go back and forth between the chemistry room and the 3D laser printer numerous times and instead integrate all steps and components into one compact tabletop machine tool.

Today, microfluidic technology is mature and readily available: The required microfluidic components such as connectors, flow switches, valves, flow controllers, switch flow matrices, etc. can all be bought off the shelf. All of these components can be handled and connected just like electronic components and cables. Microfluidics has already been used together with conventional photolithography (*30*, *31*). The challenges in regard to 3D laser lithography are rather coping with the very tight space around the high–numerical aperture (NA), high-magnification lens system of the 3D laser printer and the vastly different liquid viscosities η of, e.g., the required photoresists and solvents. These differences translate into very large gas pressure differences that are required to push the liquids through the small-diameter tubing. Furthermore, the small dimensions and narrow channels of the writing chamber imply small velocities and, hence, small Reynolds numbers for all involved liquids, leading to laminar flow. In sharp contrast, when rinsing a structure with a spray bottle by hand in the chemistry room, the liquid flow can be turbulent, which presumably often helps in getting rid of residues. The question arises: Can all process steps be performed within the regime of laminar flow? Last, such a system will consume larger quantities of all chemicals as not only the printing chamber has to be filled but also the volume of the cylindrical supply hoses connecting the chemical container to the microfluidic chamber. The inner volume *l*π*r*^2^ of the supply hoses can obviously be reduced by shortening the hose length *l*, which is limited by geometrical constraints, and by going to smaller inner tubing radii *r*. The latter step, however, increases the necessary gas pressure differences Δ*P* via Hagen Poiseuille’s law according to $\Delta P=8\dot{V}\eta l/(\pi r^{4})$ when keeping the volume flow rates $\dot{V}=dV/dt$ constant. The entrance glass window of the microfluidic chamber needs to be very thin (170 μm) due to the small free working distance of the focusing objective lens. Therefore, it cannot withstand pressure differences of more than a few bars. The next question to be answered is: Can an attractive overall system appreciating all of these constraints be realized?

Here, we present such a system. We illustrate its capabilities by a recently introduced application example, for which the need for multiple ingredient materials is immediately obvious: deterministic microstructured 3D fluorescent security features based on multiple emission colors. For this example, the recent state of the art (*16*) based on conventional systems and processes can directly be compared with the advances described in this article. We use seven different liquids within the microfluidic system: a nonfluorescent photoresist for the structure’s backbone, four photoresists containing fluorescent semiconductor quantum dots and organic dyes with different emission colors, and two developers (mr-Dev 600 and acetone). The scaling-up to a yet larger number of chemicals is straightforward.

## RESULTS AND DISCUSSION

### Microfluidic experimental setup

**Fig. 1:**
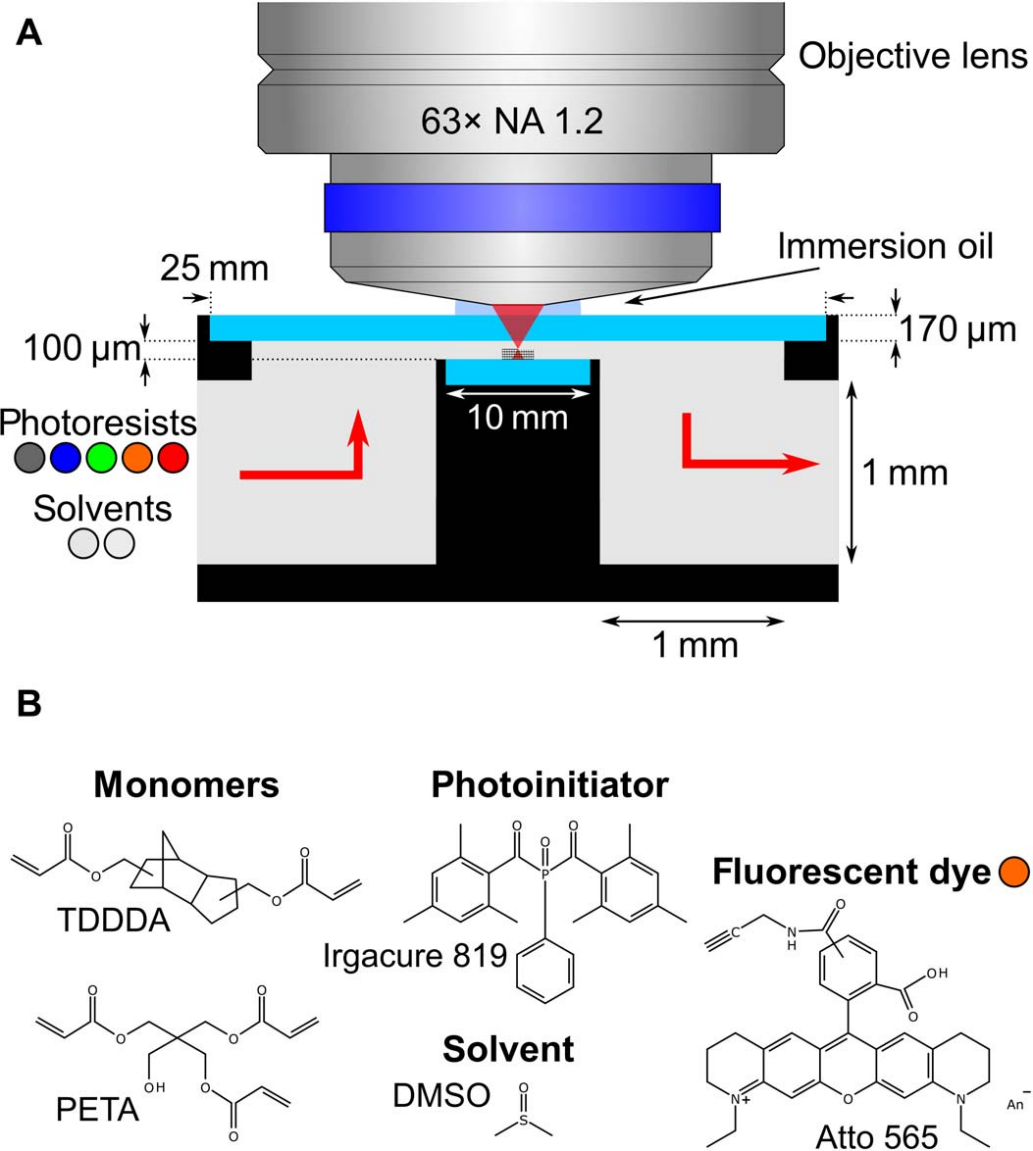
Scheme of the microfluidic chamber. (**A**) A high-NA oil-immersion microscope objective lens focuses femtosecond laser pulses into a chamber, which is clad by two thin glass windows (light blue). One of them serves as the substrate for the samples. The selection valve shown in Fig. 3 allows for switching between different photoresists (here, one nonfluorescent and four fluorescent) and solvents (acetone and mr-Dev 600), which are injected into the microfluidic chamber. For clarity, the scheme is not to scale. A to-scale technical drawing is shown in Fig. 2B. (**B**) Structure formulae of the components of one of the fluorescent photoresists containing Atto dye molecules.

**Fig. 2:**
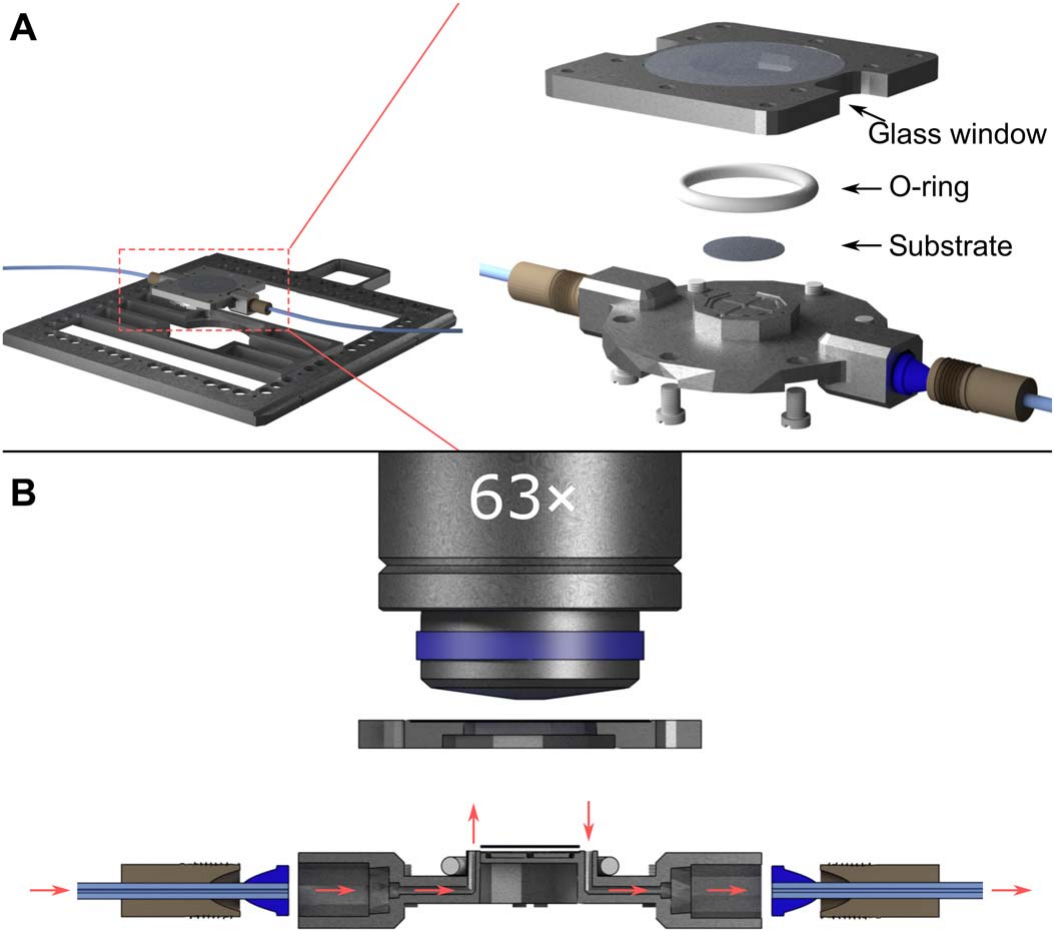
Microfluidic sample holder for 3D laser lithography. (**A**) Left-hand side: Scheme of the complete sample holder, which can be placed into a commercial 3D laser lithography machine. Right-hand side: Explosion drawing of the microfluidic chamber, which hosts a small coverslip (diameter, 10 mm) inside the chamber, onto which structures can be 3D-printed. The chamber is sealed using a solvent-resistant O-ring, and the top part features a circular glass window for the high-NA oil-immersion objective to focus inside the chamber. (**B**) Cross-sectional scale drawing of the sample holder. The sample holder features connectors for liquid tubing and channels for the liquids to be guided in and out of the microfluidic chamber. The liquid flow path is indicated using red arrows.

A scheme of the microfluidic chamber that we have built for application in combination with a commercial 3D direct laser writing system is shown in Fig. 1A. An example photoresist is shown in Fig. 1B. To understand the details, Fig. 2A shows an expanded view of the microfluidic chamber, which is made of stainless steel. Optical access is warranted by a round glass window (diameter, 25 mm; thickness, 170 μm). Another round glass window (diameter, 10 mm; thickness, 170 μm) on the opposite side of the chamber serves as the substrate onto which samples are printed. The distance between the two windows is 100 μm. This distance obviously limits the possible height of structures that can be 3D-printed. It is determined by the free working distance of the high-NA oil-immersion microscope objective lens minus the thickness of the glass window. The thickness of the glass window is limited by mechanical stability. We will further discuss this aspect below.

Because of this design, the possibilities and limitations of 3D laser lithography are the same as without using a microfluidic chamber (*28*). In particular, overhanging structures can be fabricated, the printing resolution can be tuned, and large sample footprints are possible (of course, limited by the substrate size). Likewise, optical aberrations may arise from small differences in the refractive indices of an unpolymerized photoresist and an already cross-linked material or from the difference in refractive index of the different cross-linked materials.

To insert and take out the substrate, it must be possible to reproducibly open and close the microfluidic chamber. Therefore, it consists of a top and a bottom part, as can be seen in Fig. 2A. The glass window toward the microscope objective lens is glued permanently to the top part using a two-component epoxy adhesive (UHU plus endfest 300, UHU GmbH & Co. KG), which is resistant to almost all organic solvents and (if needed) can only be dissolved by immersing it in dichloromethane overnight. Also, the top part features a groove for a solvent-resistant O-ring (14 mm by 1.78 mm; Viton FEP Encapsulated, Eastern Seals Ltd.), which seals the fluidic sample holder, making it leakproof. Apart from holding the substrate, the lower part of the sample holder also contains small channels with an inner diameter (ID) of 1 mm that guide the liquid flow in and out of the microfluidic chamber of the sample holder, as can be seen in the cross-sectional view of the sample holder in Fig. 2B (see also Fig. 1A).

**Fig. 3:**
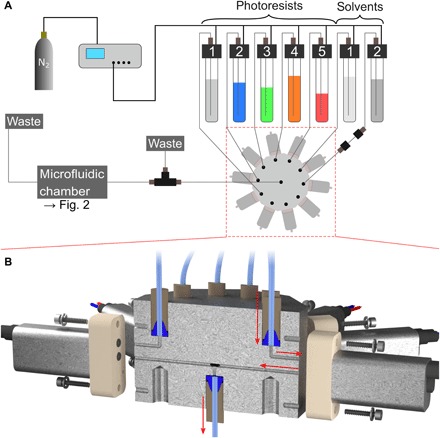
Scheme of the system connected to the microfluidic chamber. (**A**) It consists of an electronic pressure controller connected to a nitrogen bottle, up to 10 containers for the photoresists and solvents for development, and the star-shaped selection valve. Pumping individual liquids is possible by applying a pneumatic pressure to all liquid containers and opening the flow path for a single liquid using the selection valve. Following the selection valve, the liquid flow is guided through an overpressure valve and our homebuilt sample holder. Last, it is directed into a waste container. (**B**) Cross section through our homebuilt selection valve assembly. The assembly consists of commercial solenoid valves and a homebuilt 10-to-1 manifold that connects the 10 liquid containers to 10 solenoid valves, and the valve outputs to one manifold output port. An example flow path for one liquid is indicated with red arrows.

The worst-case scenario when using the microfluidic setup inside the 3D direct laser writing system is a bursting glass window caused by excessive overpressure inside the microfluidic chamber. Thus, we have taken several precautions to avoid this scenario. First, we have measured the critical overpressure at which the glass window typically fails in independent controlled combustion tests. In these tests, we usually found a critical overpressure value larger than 3 bar. Second, to reduce the overpressure inside the microfluidic chamber inasmuch as possible, we connect the output of the microfluidic chamber to the waste container using a fluorinated ethylene propylene tube with a relatively large ID of 1.0 mm. The outer diameter (OD) is 1.59 mm. Third, we never set the pressure controller to an overpressure exceeding 2 bar and feed the pressure controller only with a nitrogen overpressure slightly above 2 bar. Fourth, we have also installed a pressure relief valve (back pressure regulator P-791 and branch tee P-612, IDEX Health and Science) between the distributor valve and the input to the microfluidic chamber (see Fig. 3A). Thus far, with these precautions, the glass window has yet to fail.

In addition to the described microfluidic chamber, the overall system also includes an electronic pressure controller that is connected to a nitrogen bottle, several reservoirs containing the different photoresists and developer liquids, a homebuilt distributor valve, and tubings connecting the different components. These aspects are illustrated in Fig. 3. In particular, the system can be used to pump the different liquids as follows: Using the electronic pressure controller (one channel with 0 to 8000 mbar; Elveflow OB1 MKIII, Elvesys SAS), a defined nitrogen pressure can be applied to all liquid containers simultaneously (15-ml Falcon centrifuge tubes). The liquid containers are sealed with gas-tight lids (Elveflow) that, in addition to the inlets for the nitrogen tubes coming from the pressure controller (2.5-mm-ID polyurethane tube), have connectors for the liquid-carrying tubes. These tubes are attached to the liquid containers in such a way that they dip into the liquids inside the containers, and thus, if an overpressure is applied to the whole container, then the liquids are ejected through these tubes. Throughout the whole microfluidic system, all liquid-carrying tubes have an OD of 1.59 mm and are connected using 1/4-28 flat-bottom flangeless PEEK (polyether ether ketone) or Delrin fittings and ETFE ferrules (e.g., P-201, P-200X, or XP-235X; IDEX Health and Science).

It is possible to select and pump individual liquids due to our homebuilt distributor valve assembly, which is highlighted in Fig. 3B. Originally, we started by using a commercial high-performance liquid chromatography valve. However, after a few months of use, this valve became leaky because the sealing surfaces developed scratches due to the photoresist that polymerized on the inner surfaces. Therefore, we constructed a dedicated homebuilt distributor valve assembly. This assembly consists of 10 normally closed solenoid valves (LVM09R3Y1-5C-6-Q, SMC Corporation) connected to a homebuilt, machined aluminum 10-to-1 manifold (cross-sectional view in Fig. 3B) that guides the different liquids through the valves and eventually connects the outputs from the valves to one single output port. Internally, the wetted components of the solenoid valves consist of PEEK and Kalrez. These materials resist the different organic chemicals typically used in the workflow of 3D laser lithography. We switch the valves in a computer-controlled manner using a simple amplifier circuit incorporating a microcontroller board (Arduino Uno).

When using the microfluidic system in a 3D laser lithography setup, it is usually desirable to reduce the unnecessary consumption of photoresist as far as possible. This quantity is determined most importantly by the swept volume of all fluidic components that are connected to the input of the microfluidic chamber. Considering the small swept volume of the distributor valve (49 μl) and the fluidic chamber (25.9 μl), the major contribution stems from the tubings that are needed to connect the single components; e.g., if one would use a 1-m-long tubing with an ID of 2 mm, the swept volume would be as high as 3.1 ml. We thus aimed to reduce the ID of the tubings used as far as possible while keeping the required pump pressures at an acceptable level. As a good compromise, for the rather viscous photoresists, we ended up using connection tubings with an ID of 0.03″ = 762 μm (OD, 1.59 mm). In total, the lengths of tubing between the liquid container, the distributor valve, the pressure relief valve, and the sample holder sum up to a total length of 85 cm of tubing that liquid has to be pumped through before reaching the sample holder. Hence, for the photoresists, the tubings alone add a swept volume of 388 μl in total, resulting in a swept volume of approximately 463 μl for the whole system. Thus, we typically consume about 0.5 ml of photoresist for each injection. Compared to 3D laser lithography in a conventional writing mode, where a droplet of photoresist with a volume between 25 and 100 μl is usually sufficient in most cases, this means that photoresist consumption is increased when using the microfluidic system. For the two less viscous solvents, we used polytetrafluoroethylene tubing with an ID of 500 μm between the liquid container and the distributor valve.

In pressure-driven microfluidics, a problematic case is the one where the sample chamber is filled with a very viscous photoresist and one aims to inject a low-viscosity solvent into the sample holder. When injecting the solvent at a constant pump pressure, this would lead to low flow rates in the first moment when the system is still filled with the viscous photoresist and to excessive flow rates as soon as most of the system is filled with solvent. As an easy and very effective solution to this problem, we added a flow restrictor to the flow path of the solvent (acetone, very low dynamic viscosity η = 0.38 mPa·s), which is used to remove photoresists out of the sample holder. The flow restrictor (Elveflow SAS) consists of a PEEK capillary with a diameter of 65 μm and a length of 5 cm and leads to similar pump pressures at similar flow rates for photoresists and solvent. This can be easily understood by a simple example calculation. Consider a tube with a length of 1 m and an ID of 0.03″. Half of the tube is filled with pentaerythritol triacrylate (PETA; η = 1 Pa·s), and the other half is filled with acetone (η = 0.38 mPa·s). The acetone-filled side is connected to an acetone reservoir, and an overpressure of 1.5 bar is applied to it. Using Hagen-Poiseuille’s equation (which can be seen in direct analogy to Ohm’s law, where the tubes and the flow restrictors correspond to electrical resistors), we calculated the resulting flow rates. During the filling of the tube with acetone, for a constant applied overpressure, the flow rate would shoot up from 2.5 to 3266.4 μl/s (when the tube is completely filled with acetone). This means that, in this case, the flow rate would shoot up by a factor of 1316, which is not acceptable for any of our experiments. Now, consider the case where a flow restrictor (diameter, 65 μm; length, 5 cm) is additionally connected between the tube and the acetone reservoir. The same calculation now yields an increase in flow rate from 1.44 to 3.46 μl/s, corresponding to a moderate increase by a factor of 2.4. Thus, at the expense of elevated pumping pressures (which is acceptable for fluids with very low viscosity), the use of a flow restrictor is a very effective solution to this problem. A flow restrictor in the acetone path has been used in all of our experiments.

### Multimaterial fluorescent 3D security features

To showcase the capabilities of our system by a demanding example, we have chosen to fabricate 3D fluorescent security features similar to the ones we have already published (*19*). These security features consist of a 3D cross-grid (periods *a*_*xy*_ = 7.5 μm, *a*_*z*_ = 9 μm; lateral beam widths of approximately 0.75 μm) as a backbone, with fluorescent markers out of four different fluorescent photoresists printed into it, effectively resulting in a 3D matrix of fluorescent markers with different emission colors. In the *xy* plane, the markers are designed to have an extent of 3 μm by 3 μm. The stabilizing walls have a thickness of 5 μm. However, because of our microfluidic system, we were able to double the number of different fluorescent colors in the structure (four instead of two) and have also increased the lateral density of the markers by a factor of 2 (leading to a fourfold density of markers).

**Fig. 4:**
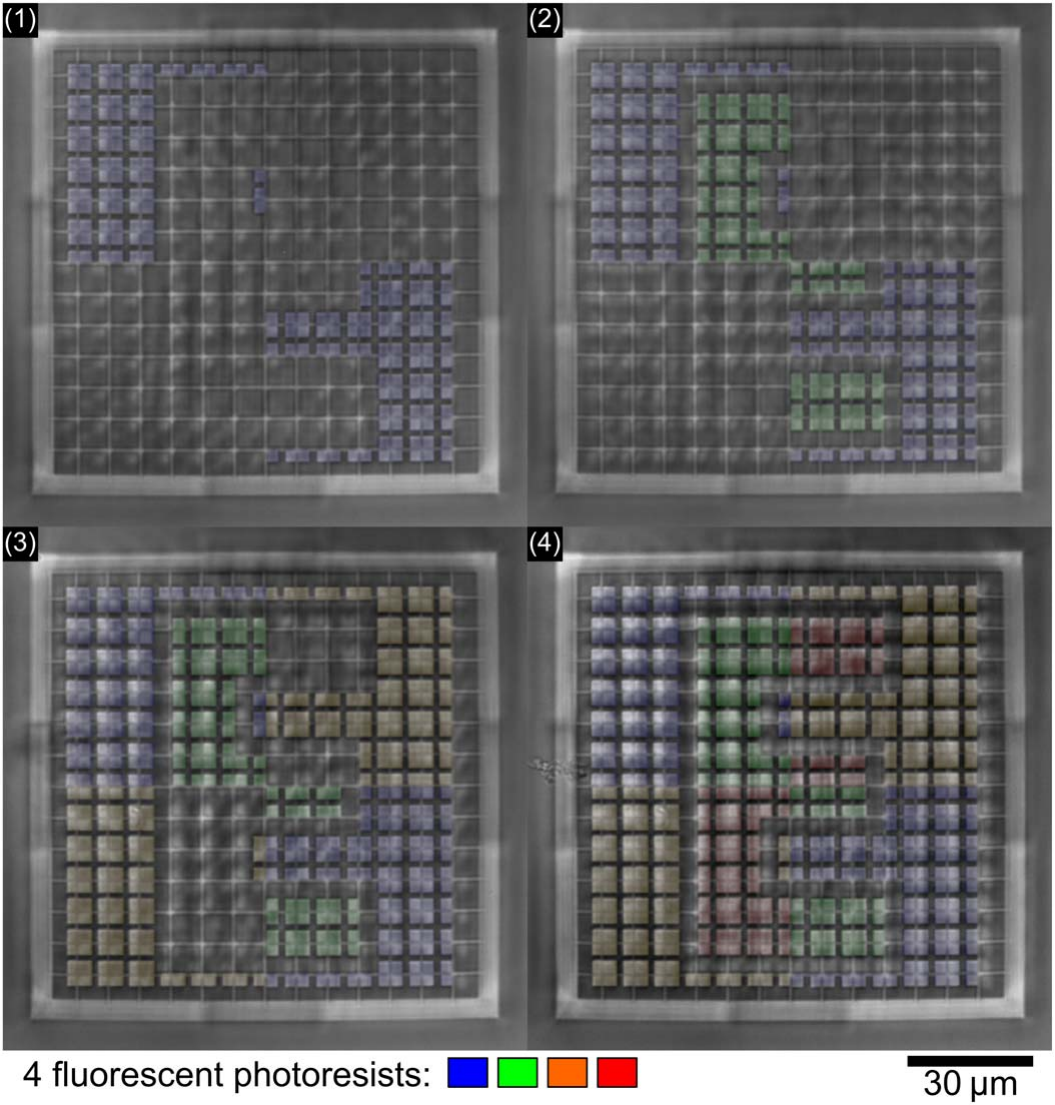
Successive 3D printing of different photoresists. Images taken using the camera integrated into our 3D laser lithography machine. Each image shows the uppermost layer of the 3D microstructure, but after different printing steps. For the first picture, the 3D support grid and blue fluorescent markers have been printed, whereas for the last picture, markers using all four fluorescent resists have been printed. For clarity, fluorescence emission colors are overlaid.

In the fabrication workflow, we first inject the nonfluorescent photoresist into the microfluidic chamber. As this photoresist is rather viscous, we use an overpressure of Δ*P* = 2000 mbar for *t* = 120 s. After the 3D support grid has been printed, we develop the sample by injecting acetone (Δ*P* = 1500 mbar, *t* = 60 s) and mr-Dev 600 (Δ*P* = 150 mbar, *t* = 60 s) consecutively. We then 3D-print the fluorescent parts of the structure by repeatedly injecting the fluorescent photoresists, printing, and developing with acetone and mr-Dev 600 as described above. The blue- and green-emitting photoresists contain CdSeS/ZnS quantum dots (Δ*P* = 500 mbar, *t* = 45 s), whereas the orange- and red-emitting resists (Δ*P* = 1000 mbar, *t* = 40 s) contain organic Atto dyes (see Materials and Methods for further details). Images of the top layer of a written structure taken using the camera integrated into the 3D laser lithography system are depicted in Fig. 4. Each image was taken after the printing of a new fluorescent photoresist was finished, and the false-color overlays correspond to the different fluorescence emission colors of the different parts of the structure. After finishing the last printing and development step, the sample is finished and no further process steps are required. However, to clean up the sample, we typically perform an additional cleaning step by rinsing the sample in acetone and isopropanol and carefully blow-drying the sample with a nitrogen gun.

**Fig. 5:**
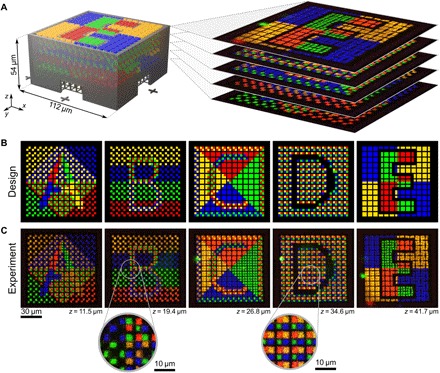
Confocal laser scanning fluorescence microscopy of fabricated structures. (**A**) On the left-hand side, a computer rendering of the design for the microstructure is shown. It consists of a nonfluorescent 3D support structure (gray) with fluorescent markers with different emission colors printed into it. On the right-hand side, a stack of images taken by using fluorescence microscopy is shown. (**B**) The designs of the test patterns were printed into the five different marker layers of the microstructure. (**C**) Measurement data from fabricated microstructures taken using fluorescence microscopy. Insets show the level of detail at which different photoresist structure elements can be printed.

The left-hand side of Fig. 5A shows the computer design of the 3D fluorescent security feature. It consists of the 3D cross-grid surrounded by walls for support depicted in gray, with four fluorescent markers arranged laterally around every grid point. In total, this results in 26 × 26 × 5 possible marker positions in the *x*, *y*, and *z* direction. Thus, around 7.8 kbit of information can be stored inside the security feature. For characterization of the fabricated structures, we use confocal laser scanning microscopy (LSM), which we use to image the different fluorescent parts in three dimensions (see Materials and Methods for details). The right-hand side of Fig. 5A shows how the different *z*-layers of markers are arranged inside the 3D microstructure. The computer designs of the test patterns we printed inside the security features are shown in Fig. 5B, and the measured images of single marker layers of an actual fabricated microstructure taken using LSM are depicted in Fig. 5C. The intensity normalization is the same for all fluorescence images shown. In addition, insets in Fig. 5C show the level of detail at which the fluorescent parts of the structure are printed. As can be seen in the figure, the agreement between the designed test patterns and the measured data is excellent.

## CONCLUSION

We introduce a microfluidic system that can perform all photoresist injection and sample development steps inside the laser lithography machine. Thus, our system heavily facilitates the fabrication of 3D laser lithography structures consisting of multiple materials. To highlight the capabilities of our system by example, we have printed complex 3D security features, which consist of five different photoresists, in total using seven different liquids for fabrication inside the laser lithography system. It is conceivable that these microfluidic systems will become widely established for the manufacture of complex 3D micro- and nanostructures composed of multiple materials, with applications in diverse fields such as 3D scaffolds for cell culture, 3D metamaterials, 3D micro-optical systems, and 3D security features. As we have shown, the system can even be integrated into commercially available state-of-the-art 3D laser lithography machine tools.

## MATERIALS AND METHODS

### Materials

All chemicals were used without further purification. The materials used (and their corresponding manufacturers) were as follows: acetone (99.5%; Sigma-Aldrich), Atto 565 (alkyne functionalized; ATTO-TEC GmbH), Atto 647N (alkyne functionalized; ATTO-TEC GmbH), Irgacure 819 (Ciba Inc.), dimethyl sulfoxide (DMSO; 99%; Merck), isopropanol (99.5%; Sigma-Aldrich), PETA (technical grade; Sigma-Aldrich), mr-Dev 600 (micro resist technology GmbH), toluene (99.8%; Sigma-Aldrich), tricyclo[5.2.1.0^2,6^]decanedimethanol diacrylate (TDDDA; technical grade; Sigma-Aldrich), Trilite Fluorescent Nanocrystals 450 nm (alkyl functionalized; Cytodiagnostics), Trilite Fluorescent Nanocrystals 525 nm (alkyl functionalized; Cytodiagnostics), and 3-(trimethoxysilyl)propyl methacrylate (98%; Sigma-Aldrich).

### Nonfluorescent photoresist

The photoresist was obtained by mixing the multifunctional monomer PETA with 2% (w/w) Irgacure 819 as photoinitiator and subsequent treatment for 30 min in an ultrasonic bath.

### Photoresists containing quantum dots

First, 4% (w/w) of Irgacure 819 was added to PETA and ultrasonicated until the photoinitator was dissolved completely (mixture A). We then blended mixture A with the nonpolar monomer TDDDA and toluene in a volume ratio of 1:1:2 (mixture B). While stirring on a magnetic stirrer, we slowly added 10% (w/w) nonpolar (oleic acid) functionalized CdSeS/ZnS alloyed quantum dots in toluene solution (1 mg/ml) to mixture B. Depending on the desired fluorescence emission color, we used either blue- or green-emitting quantum dots (λ_em_ = 450 nm or λ_em_ = 525 nm). Last, we evaporated toluene from the photoresist while stirring at 35°C for 20 hours. After evaporation, the photoresists still contained about 10% (v/v) toluene.

### Photoresists containing Atto dyes

Atto 647N and Atto 565 (alkyne functionalized) were dissolved in DMSO at a concentration of 0.1 mg/ml. Then, 4% (w/w) Irgacure 819 was dissolved in PETA. TDDDA was added in a volume ratio of 1:1. We finally added 10 μM Atto dye in DMSO and stirred the mixture for 5 min. The final photoresists contain about 6% (v/v) DMSO.

### Silanization of coverslips

To improve surface adhesion of the printed structures to the substrate, we silanized the coverslips by activating the glass surface using plasma etching for 30 min and immersing them into 1 mM solution of 3-(trimethoxysilyl)propyl methacrylate in toluene afterward. Next, the coverslips were rinsed with isopropanol and blow-dried with a nitrogen gun.

### 3D laser lithography

3D laser lithography was performed using a commercial direct laser writing system (Photonic Professional GT, Nanoscribe GmbH). This instrument includes an automated interface finder. For writing, we used a long-distance oil-immersion objective (Carl Zeiss LD LCI Plan-Apochromat 63×/1.2 Imm Korr DIC M27) with a correction ring for the refractive index of the immersion medium. Adjustment of the correction ring was performed by minimizing the writing threshold power for the nonfluorescent photoresist. To write the 3D cross-grid support structure, we typically used a constant writing laser power of *P* = 34.5 mW and a writing velocity of *v* = 1.5 cm/s. To write the fluorescent photoresists, we used a speed of 1 cm/s and a writing power of 42.5 mW for the deepest layer of fluorescent markers inside the structure and linearly decreased the writing power to 35 mW to write the uppermost layer of markers.

### Confocal LSM

We performed confocal LSM using a commercial system (LSM510 Meta, Carl Zeiss). For readout of the sample, we used an oil-immersion objective (Plan-Apochromat 63×/1.40, Carl Zeiss). For this purpose, we put the immersion oil directly onto the sample. The microscope is equipped with two photomultiplier tubes, so that two color channels can be read out simultaneously. Thus, for samples emitting at four different fluorescence colors, each *z*-layer has to be read out twice using different filter sets. We chose the following alternating routine: For each *z*-layer, we first read out the blue and the orange channel using a chromatic beam splitter at λ_BS_ = 490 nm and two band-pass filters with 420 nm < λ_BP1_ < 480 nm and 575 nm < λ_BP2_ < 615 nm. Here, the sample was excited with a power of 236 μW at 405 nm and with a power of 109 μW at 488 nm. To scan the green and the red channel, we used a chromatic beam splitter at λ = 565 nm, a band-pass filter transparent between 505 nm < λ_BP3_ < 550 nm for the green channel, and a long-pass filter with λ_LP_ > 650 nm for the red channel. For excitation, we used a blue laser (λ = 405 nm, *P* = 236 μW) and a red laser (λ = 633 nm, *P* = 28 μW). All excitation powers were measured in the back focal plane of the objective lens.

## Supplementary Material

http://advances.sciencemag.org/cgi/content/full/5/2/eaau9160/DC1

## SUPPLEMENTARY MATERIALS

Supplementary material for this article is available at http://advances.sciencemag.org/cgi/content/full/5/2/eaau9160/DC1 Fig. S1. Photograph of the microfluidic setup. Fig. S2. Photographs of the microfluidic sample holder. Movie S1. Animation of scan through different *z*-positions of the fluorescent 3D microstructure.
