# Supplementary material for: Multimaterial 3D laser microprinting using an integrated microfluidic system
Source: Sci Adv. 2019 Feb 8;5(2):eaau9160. doi: 10.1126/sciadv.aau9160 (PMC6368435; doi:10.1126/sciadv.aau9160)
Supplement: http://advances.sciencemag.org/cgi/content/full/5/2/eaau9160/DC1 [file supp_5_2_eaau9160__index.html]

Science Advances | Science Advances

## Supplementary Materials

**The PDF file includes:**

- Fig. S1. Photograph of the microfluidic setup.
- Fig. S2. Photographs of the microfluidic sample holder.
- Legend for movie S1

Download PDF

**Other Supplementary Material for this manuscript includes the following:**

- Movie S1 (.mp4 format). Animation of scan through different *z*-positions of the fluorescent 3D microstructure.

**Files in this Data Supplement:**

- Adobe PDF - aau9160\_SM.pdf
