# Supplementary material for: Multimaterial 3D laser microprinting using an integrated microfluidic system
Source: Sci Adv. 2019 Feb 8;5(2):eaau9160. doi: 10.1126/sciadv.aau9160 (PMC6368435; doi:10.1126/sciadv.aau9160)
Supplement: http://advances.sciencemag.org/cgi/content/full/5/2/eaau9160/DC1 [file aau9160_SM.pdf]

Science Advances
AAAS

advances.sciencemag.org/cgi/content/full/5/2/eaau9160/DC1

Supplementary Materials for

Multimaterial 3D laser microprinting using an integrated microfluidic system

Frederik Mayer*, Stefan Richter, Johann Westhauser, Eva Blasco, Christopher Barner-Kowollik, Martin Wegener

*Corresponding author. Email: frederik.mayer@kit.edu

Published 8 February 2019, *Sci. Adv.* **5**, eaau9160 (2019)
DOI: 10.1126/sciadv.aau9160

The PDF file includes:

Fig. S1. Photograph of the microfluidic setup.
Fig. S2. Photographs of the microfluidic sample holder.
Legend for movie S1

Other Supplementary Material for this manuscript includes the following:

(available at advances.sciencemag.org/cgi/content/full/5/2/eaau9160/DC1)

Movie S1 (.mp4 format). Animation of scan through different **z**-positions of the fluorescent 3D microstructure.

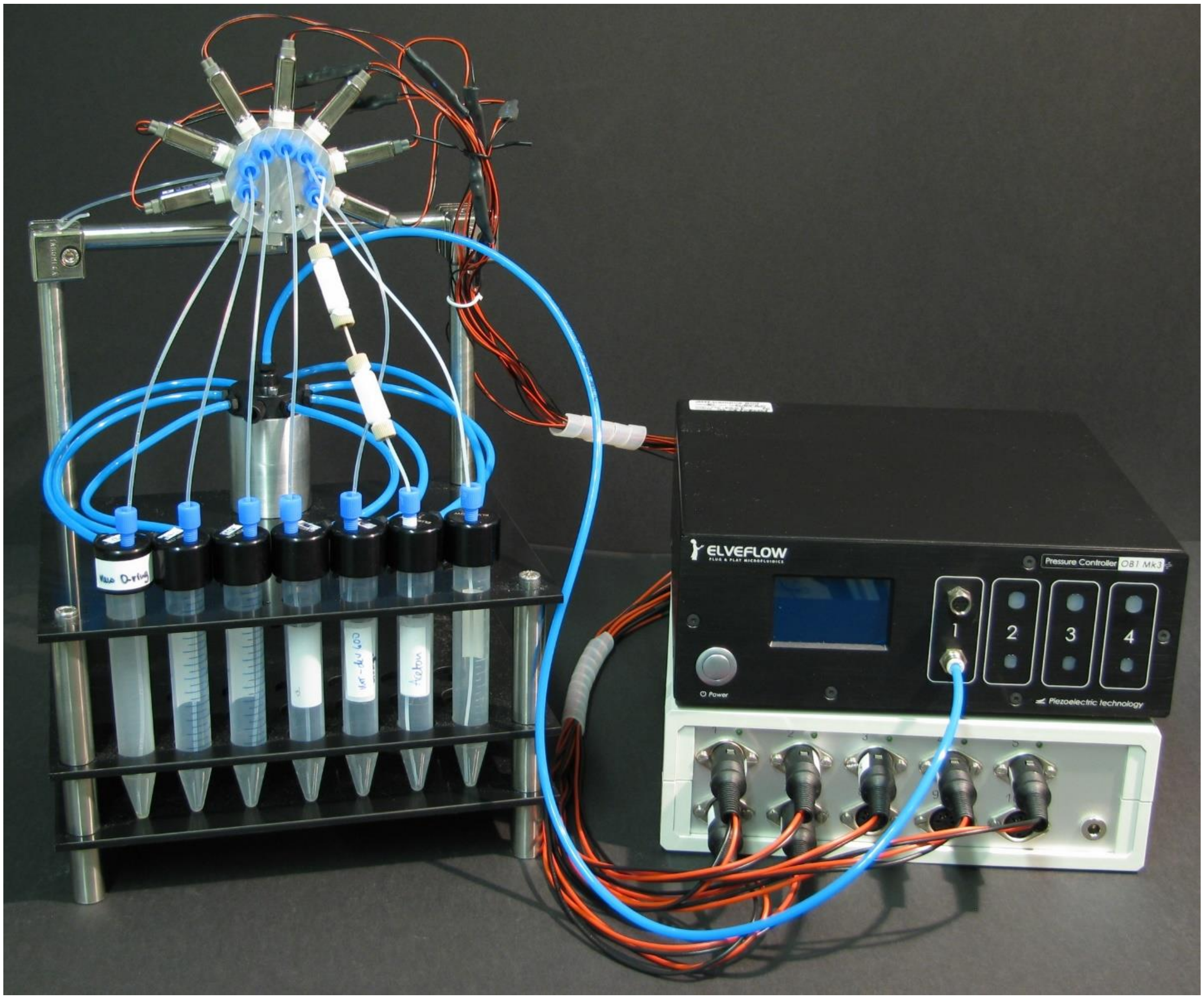

Fig. S1. Photograph of the microfluidic setup. The setup consists of an electronic gas pressure controller (black box), which is used to equally pressurize the liquid containers (via blue hoses). Pumping single liquids is possible by opening single valves of our home-built solenoid valve assembly (star-shaped part). The gray box is for controlling all solenoid valves via a computer. (Photo credit: Frederik Mayer, INT/KIT)

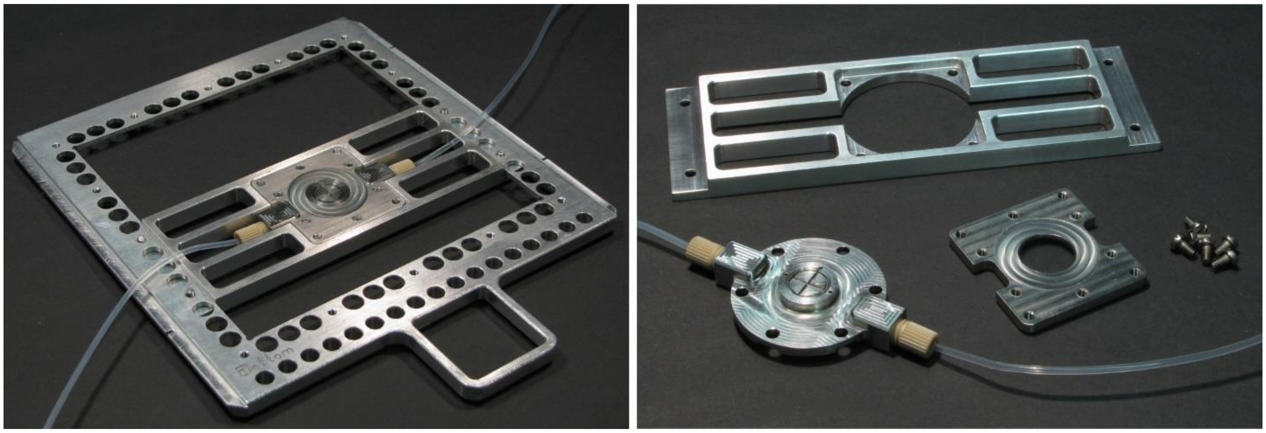

Fig. S2. Photographs of the microfluidic sample holder. (Photo credit: Frederik Mayer, INT/KIT)

Movie S1. Animation of scan through different *z*-positions of the fluorescent 3D microstructure. Images for the movie were taken using confocal laser scanning microscopy and no interpolation was performed.
